# Inhibition of Xanthine Oxidase Protects against Sepsis-Induced Acute Kidney Injury by Ameliorating Renal Hypoxia

**DOI:** 10.1155/2022/4326695

**Published:** 2022-07-15

**Authors:** Ting-ting Wang, Yi-wei Du, Wen Wang, Xiang-nan Li, Hong-bao Liu

**Affiliations:** Department of Nephrology, Tangdu Hospital, Air Force Military Medical University (Fourth Military Medical University), Xi'an 710038, China

## Abstract

Xanthine oxidase (XO) utilizes molecular oxygen as a substrate to convert purine substrates into uric acid, superoxide, and hydrogen peroxide, which is one of the main enzyme pathways to produce reactive oxygen species (ROS) during septic inflammation and oxidative stress. However, it is not clear whether XO inhibition can improve sepsis-induced renal hypoxia in sepsis-induced acute kidney injury (SI-AKI) mice. In this study, pretreatment with febuxostat, an XO-specific inhibitor, or kidney knockdown of XO by shRNA in vivo significantly improved the prognosis of SI-AKI, not only by reducing the levels of blood urea nitrogen, serum creatinine, tumor necrosis factor-*α*, interleukin-6, and interleukin-1*β* in peripheral blood but also by improving histological damage and apoptosis, reducing the production of ROS, and infiltrating neutrophils and macrophages in the kidney. More importantly, we found that pharmacological and genetic inhibition of XO significantly improved renal hypoxia in SI-AKI mice by a hypoxia probe via fluorescence staining. This effect was further confirmed by the decrease in hypoxia-inducible factor-1*α* expression in the kidneys of mice with pharmacological and genetic inhibition of XO. In vitro, the change in XO activity induced by lipopolysaccharide was related to the change in hypoxia in HK-2 cells. Febuxostat and XO siRNA significantly relieved the hypoxia of HK-2 cells cultured in 2% oxygen and reversed the decrease in cell viability induced by lipopolysaccharide. Our results provide novel insights into the nephroprotection of XO inhibition in SI-AKI, improving cell hypoxia by inhibiting XO activity and reducing apoptosis, inflammation, and oxidative stress.

## 1. Introduction

Sepsis is the leading cause of acute renal injury (AKI) and is associated with increased morbidity and mortality in intensive care units [1, 2]. Palliative interventions such as fluid resuscitation, antibiotics, vasopressin, and renal replacement therapy are recommended to treat sepsis-induced AKI (SI-AKI) patients and wait passively for the kidney function to recover [3–5]. The experimental AKI induced by lipopolysaccharide (LPS), a key component of the outer membrane of gram-negative bacteria, is commonly used in vivo model closely recapitulating SI-AKI in humans, which hosts a complex inflammatory milieu comprising neutrophils, macrophages, epithelial cells, reactive oxygen species (ROS), proinflammatory mediators, and enzymes [6, 7]. An in-depth understanding of the SI-AKI pathophysiology is of great benefit to formulating effective mechanism-mediated treatment strategies.

Xanthine oxidase (XO) is one of the main enzyme pathways that produce ROS during oxidative stress and inflammation. It utilizes molecular oxygen to catalyze the oxidation of purine substrates (such as hypoxanthine and xanthine) to uric acid and generates superoxide (O_2_^•-^) and hydrogen peroxide (H_2_O_2_) [8, 9]. XO has been suggested to participate in the pathogenesis of acute organ injury, including SI-AKI. Its activity and expression can be upregulated by various inflammatory stimuli, such as LPS, cytokines, and hypoxia [10–16]. Febuxostat, a selective and potent inhibitor of XO, can alleviate AKI, which may be related to antioxidant stress, anti-inflammation, and antiendoplasmic reticulum stress and reducing uric acid production [17–19]. The protective role of febuxostat in animal models of SI-AKI has only recently been reported. Ramos and his colleagues [20] found that febuxostat, rather than allopurinol, improved renal function in experimental SI-AKI animals induced by LPS. The mechanism may be associated with antioxidant, anti-inflammatory, and antiapoptotic effects. Similarly, Ibrahim et al. [21] confirmed the protective effect of febuxostat on liver and kidney injuries in sepsis after cecal ligation through its antioxidant, anti-inflammatory, and antiapoptotic properties and the weakening of the c-Jun N-terminal kinase signaling pathway.

In addition to the inflammatory cascade and oxidative stress, renal ischemia/hypoxia is emerging as a common pathophysiological feature of SI-AKI, an essential driver in the transition and/or propensity for the progression from AKI to chronic kidney disease (CKD) [22, 23]. Based on the characteristic that XO utilizes molecular oxygen to catalyze purine substrates, we hypothesize that downregulation of XO can alleviate local hypoxia in renal tissue of the SI-AKI model, which has not been reported thus far. Therefore, this study is aimed at exploring the effects and potential mechanisms of XO inhibition on LPS-induced renal hypoxia in SI-AKI mice.

## 2. Materials and Methods

### 2.1. Chemicals and Reagents

LPS (Escherichia coli serotype O55:B5, L2880) was obtained from Sigma-Aldrich (USA). F4/80 (cat no. 29414-1-AP) and myeloperoxidase (MPO, cat no. 22225-1-AP) antibodies were from Proteintech (Wuhan, China), and hypoxia-inducible factor-1*α* (HIF-1*α*) antibody (cat no. BF8002) was from Affinity Biologicals (Jiangsu, China). XO (cat no. sc-398548) antibody was from Santa Cruz (USA). The XO activity assay kit (cat no. KTB1070), goat anti-mouse IgG H&L (DyLight 649) (cat no. A23610), goat anti-mouse IgG H&L (DyLight 488) (cat no. A23210), and goat anti-mouse IgG HRP (cat no. A21010) were purchased from Abbkine (Wuhan, China). Pimonidazole HCl and anti-pimonidazole mouse antibody was purchased from Hypoxyprobe (USA). ELISA kits for tumor necrosis factor-*α* (TNF-*α*, cat no. BMS607-3), interleukin-6 (IL-6, cat no. KMC0061), and IL-1*β* (cat no. KMC0061) were from Thermo Fisher Scientific (USA). The fluorescein (FITC) TUNEL Cell Apoptosis Detection Kit was from Servicebio (cat no. G1501, Wuhan, China). Dihydroethidium (DHE) for probing superoxide radicals was purchased from Beyotime (cat no. S0063, Shanghai, China).

### 2.2. Animals and Experimental Protocol

All animal experiments were conducted in strict accordance with the Guidelines for Care and Use of Laboratory Animals and were permitted by the Animal Welfare and Ethics Institution of the Fourth Military Medical University. Male C57BL/6 mice aged 6-8 weeks, weighing 20-25 g, were raised in the SPF laboratory of the Animal Experiment Center of the Air Force Military Medical University (Fourth Military Medical University). They were randomly divided into three groups: control (received water by gavage) group (*n* = 10), LPS (10 mg/kg dissolved in sterile deionized water) only group (*n* = 10), and LPS+febuxostat (Feb, 10 mg/kg/day dissolved in saline) (*n* = 10). The mice were given febuxostat by gavage every 24 h for 7 days, followed by intraperitoneal injection of LPS. For XO knockdown in vivo, we constructed an AAV vector carrying XO shRNA (pAAV-shXO) with a target sequence of 5′ AAGTGTAGCAATCGCGTCC 3′ (Shanghai Genechem Co., Ltd). Mice were divided into two groups: LPS+pAAV-shNC (LPS+Ctrl-shR) and LPS+pAAV-shXO (LPS+XO-shR) (*n* = 10). AAV injection was conducted according to the previous research [24]. Briefly, mice were anesthetized with 50 mg/kg sodium pentobarbital, the abdominal hair of the mice was removed with a shaver, and the mice were fixed in a supine position on the operating table with tape. Make a longitudinal incision about 2.0 cm long along the midline of the abdomen below the costal margin. Then, renal vein was isolated and clamped, and 50 *μ*l of 1× PBS containing 1*E* + 11 V.G. of AAV was slowly injected using a 30 G needle. The clamp was removed after 15 min postinjection, and the incision sutured. Three weeks later, LPS was administered to induce SI-AKI in mice. Animals were ethically sacrificed by administrating pentobarbital sodium (Sigm-Aldrich, USA) at 24 h after LPS injection, and whole blood and kidneys were collected for further analysis.

### 2.3. Blood Physiochemical Assays

The whole blood collected from the eyeballs was centrifuged at 4°C and 4000 rpm for 10 min to acquire the serum sample. The levels of serum creatinine (Scr, cat no. C011-2-1) and blood urea nitrogen (BUN, cat no. C013-2-1) were measured according to the manufacturer's instructions using the creatinine and urea nitrogen assay kit (Nanjing Jiancheng, China).

### 2.4. Renal Histopathology

Kidney tissues were carefully separated, washed with ice-cold stroke-physiological saline solution, and stored in 4% paraformaldehyde. Hematoxylin and eosin (H&E) staining of paraffin-embedded kidney tissue slices was performed, and a double-blind method was utilized to assess the damage to renal tubular epithelial cells. H&E-stained sections were scored by calculating the percentage of tubules in corticomedullary junction that displayed cell necrosis, loss of brush border, cast formation, and tubular dilation as follows: 0, none; 1, <10%; 2, 11-25%; 3, 26-45%; 4, 46-75%; and 5, >76%. At least 10 randomly selected areas per mouse were assessed. The scores of ten fields per kidney section were averaged and used as the score of individual mouse kidneys.

### 2.5. Determination of XO Activity and Expression in Serum and Kidneys

After renal tissue homogenization, kidney XO activity was detected according to the manual instructions, and the XO activity in the serum was detected at the same time. XO expression in renal tissue was further detected by immunofluorescence. Briefly, kidney tissues were sliced into 5 *μ*m sections, stained with XO antibody (1 : 100) for 18 h at 4°C, washed with phosphate buffered saline with Tween 20 (PBST), incubated with goat anti-mouse IgG (DyLight 488) (1 : 3000), and stained for 1 h at room temperature in the dark. After washing with PBST, DAPI solution was added to stain the nucleus, and photos were taken with a laser confocal microscope (Leica SP8).

### 2.6. Determination of Hypoxia in Kidneys

Hypoxyprobe, the component of which is pimonidazole hydrochloride, has been an effective approach to assessing hypoxia in cells [25]. Pimonidazole hydrochloride could be reduced, activated, and combined with thiol groups from peptides or proteins which could be detected with goat anti-mouse hypoxyprobe antibody. Twenty-four hours after the SI-AKI model was established, and the mice were injected intraperitoneally with 60 mg/kg hypoxyprobe and anesthetized with 50 mg/kg sodium pentobarbital 2 h later. The kidney tissues were collected and stored at -80°C. Kidney tissues were sliced into 5 *μ*m sections, stained with goat anti-mouse hypoxyprobe antibody (1 : 200) for 18 h at 4°C. Kidney slices were washed with PBST; then, goat anti-mouse IgG (DyLight 649) (1 : 3000) was added and stained for 1 h at room temperature in the dark. After washing with PBST, DAPI solution was added to stain the nucleus, and photos were taken with a laser confocal microscope (Leica SP8).

### 2.7. Western Blot Analysis

Equal amounts of protein from HK-2 cells or kidney tissue lysates were loaded and separated using 10% sodium dodecyl sulfate (SDS) polyacrylamide gels and transferred to polyvinylidene fluoride membranes (cat no. IPVH00010, Millipore, USA). The membranes were incubated with 5% nonfat milk for 1 h at room temperature and probed with HIF-1*α* or XO primary antibody for 18 h at 4°C, followed by a peroxidase-conjugated secondary antibody. Antibody-antigen complexes were detected using an ECL system (cat no. P0018AS, Beyotime, Shanghai, China). The intensity of each band was measured using ImageJ. The results were normalized to the intensity of beta-actin for standardization.

### 2.8. ROS Detection in Kidneys

Dihydroethidium (DHE) was used to detect the ROS levels in the renal tissues, as previously reported [26]. The renal tissues were immersed in saccharose (30% w/v), embedded at the optimal cutting temperature (OCT), sliced into 5 *μ*m sections, and stored at -20°C until fluorescence detection. Tissue sections were incubated with 10 *μ*M DHE for 60 min at 37°C in a humidified chamber in the dark, incubated with DAPI solution at room temperature for 5 min, and kept in the dark. In the presence of superoxide anions, DHE is oxidized to ethidium, producing bright red fluorescence. After washing with PBS, sections were visualized and imaged via a laser confocal microscope (Leica SP8).

### 2.9. Macrophages and Neutrophils Infiltrated the Kidney Tissues

Tissues were fixed with 4% paraformaldehyde and subsequently processed for immunofluorescence staining. MPO and F4/80 staining was performed after antigen retrieval (1% SDS for 3 min). MPO^+^ and F4/80^+^ cells were quantified by counting the number of stained cells per field. We collected 10-15 images of a kidney from each animal at 400x magnification with a laser confocal microscope (Leica SP8).

### 2.10. Cytokine Analysis

The concentrations of the cytokines TNF-*α*, IL-6, and IL-1*β* in serum were measured with mouse TNF-*α*, IL-6, and IL-1*β* ELISA kits according to the instructions.

### 2.11. Kidney Terminal Deoxynucleotidyl Transferase dUTP Nick-End Labeling (TUNEL) Assay

Kidney tissue TUNEL analysis was conducted in accordance with our previous research [27]. Briefly, kidney tissues were fixed with 4% paraformaldehyde (PFA) for 24 h at room temperature, followed by dehydration and paraffin embedding. Tissues were cut into 5 *μ*m sections for immunofluorescence staining. The sections were incubated with TdT enzyme solution for 90 min at 37°C. Then, FITC-12-dUTP was added and incubated for 30 min at 37°C. The reaction was terminated by incubation in stop/wash buffer for 30 min at 37°C. The number of TUNEL-positive cell nuclei and the total numbers of cell nuclei stained with DAPI were counted in 10 random areas, and the percentages of the numbers of TUNEL-positive nuclei to the numbers of total cell nuclei were then calculated.

### 2.12. Cells and Experimental Protocol

Cell culture experiments were performed using HK-2 cells, a human kidney proximal tubular cell line, which was purchased from the American Type Culture Collection. HK-2 cells at a concentration of 5 × 10^3^/well in 96-well plates were cultured with or without LPS (10 *μ*g/ml) in a trigas incubator (Thermo Fisher Scientific, USA) under 21% O_2_ or 2% O_2_ for 6 h in the presence or absence of febuxostat (100 *μ*M). Then, we added 10 *μ*L CCK-8 (Dojindo, Japan) to each well and measured the optical density (OD) values at 450 nm after 1 h of incubation. For hypoxia condition evaluation, HK-2 cells were thus divided into three groups: control, LPS (10 *μ*g/ml), and LPS (10 *μ*g/ml) plus febuxostat (100 *μ*M). After administration of LPS and febuxostat, cells were incubated for 6 h. For XO knockdown analysis, HK-2 cells were transfected with siRNA of XO (XO-siR) or negative control (Ctrl-siR) using Lipofectamine® 2000 (Invitrogen) according to the manufacturer's protocol. The target sequence of si-XO was 5′-GGCATTGAGATGAAGTTCA-3′. The oligonucleotide dose used was 100 nM. All transfections were transient. Thirty-six hours later, the cells were treated with LPS (10 *μ*g/ml) with or without 2% oxygen. Then, 150 *μ*M pimonidazole HCl was added to the cells 1 h before being harvested for hypoxia evaluation, after fixation with 4% paraformaldehyde (PFA) for 20 min at room temperature. Anti-pimonidazole mouse antibody was incubated with the cells at 4°C for 18 h. Afterward, goat anti-mouse DyLight 488 antibody was added and incubated for 1 h at room temperature in the dark. Finally, confocal imaging was conducted with a Leica SP8 confocal microscope. For XO activity analysis, HK-2 cells at a concentration of 5 × 10^4^/well in 6-well plates were cultured with or without LPS (10 *μ*g/ml) in a trigas incubator under 21% O_2_ or 2% O_2_ for 6 h in the presence or absence of febuxostat (100 *μ*M) or siXO transfection. After that, the cells were harvested for the XO activity assay conducted according to the assay kit instructions.

### 2.13. Statistical Analysis

Data are presented as the mean ± standard deviation (SD). Differences between different data means were compared by using Student's *t*-test and one-way analysis of variance (ANOVA) followed by Dunnett's multiple comparison tests using GraphPad Prism 7. *P* < 0.05 indicates that the difference is statistically significant.

## 3. Results

### 3.1. Effects of Febuxostat on Attenuating Renal Injury in SI-AKI Mice

**Figure 1:**
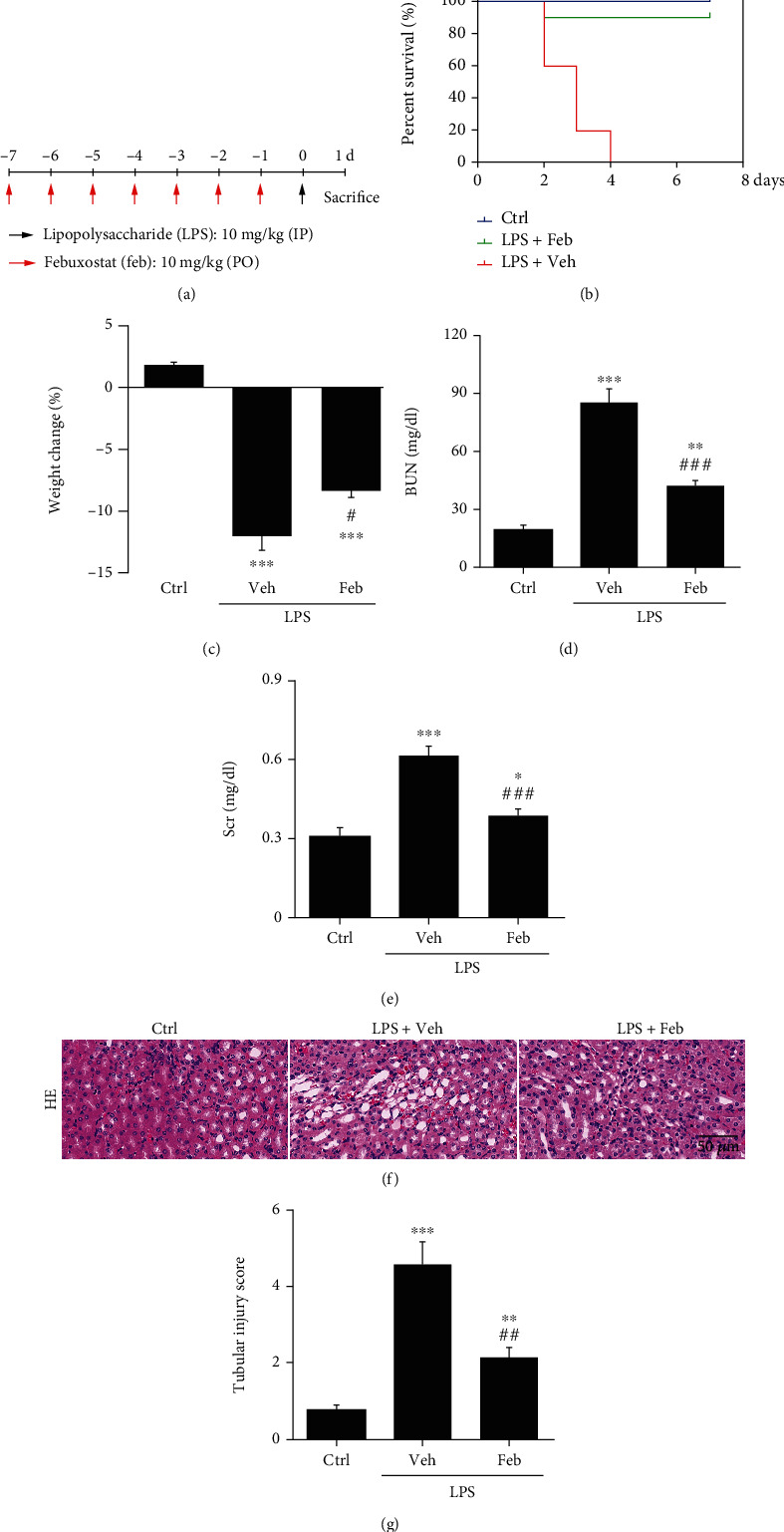
Therapeutical effects of febuxostat on attenuating renal injury in SI-AKI mice. (a) Mice pretreated with febuxostat (10 mg/kg/day, po.) for 7 days were administered intraperitoneal injections of LPS (10 mg/kg) and then were executed 24 h after LPS injection. (b) The death of mice in 7 days after intraperitoneal injection of LPS (10 mg/kg). (c–e) Body weight changes and BUN and Scr levels were measured at 24 h after the injection of LPS. (f, g) Histopathology analysis of the kidneys in SI-AKI mice was performed by hematoxylin-eosin (HE) staining (600x magnification), and the kidney tubular injury score was graded in a double-blinded manner. Scale bar = 50 *μ*m. ^∗^*P* < 0.05, ^∗∗^*P* < 0.01, and ^∗∗∗^*P* < 0.001 vs. Ctrl; ^#^*P* < 0.05, ^##^*P* < 0.01, and ^###^*P* < 0.001 vs. LPS+Veh (*n* = 10). Ctrl: control; Veh: vehicle; LPS: lipopolysaccharide; Feb: febuxostat; BUN: blood urea nitrogen; Scr: serum creatinine.

The schedule of LPS (10 mg/kg, ip.) and febuxostat (Feb, 10 mg/kg/day, po.) administration was outlined (Figure 1(a)). We first administered febuxostat daily one week before establishing the LPS-challenged SI-AKI model. We recorded the survival of SI-AKI mice with or without febuxostat pretreatment within one week (Figure 1(b)). Control mice exhibited 100% survival. Forty-eight hours after LPS administration, 40% of septic mice died, and after another 48 h, the remaining septic mice died. In contrast, febuxostat pretreatment dramatically increased the survival rate by 90% compared to the LPS group (Figure 1(b)). Therefore, the SI-AKI mice were killed 24 h after LPS injection for the subsequent experiments.

Febuxostat pretreatment showed remarkable resistance to weight loss in SI-AKI mice (Figure 1(c)). Induction of sepsis by LPS caused a significant increase in blood urea nitrogen (BUN, Figure 1(d)) and serum creatinine (Scr, Figure 1(e)) compared to the control group. Pretreatment with febuxostat exerted a significant decrease in BUN and Scr levels compared to the LPS group (Figures 1(d) and 1(e)). The specific histopathological features of SI-AKI showed that many renal tubular epithelial cells were vacuolated, the brush border was lost and flattened, and protein cast was also observed (Figure 1(f)). After febuxostat pretreatment, renal tubular epithelial cell injury was significantly improved (Figure 1(g)).

### 3.2. Febuxostat Relieves Serum and Renal Tissue XO Activity, and XO Knockdown Attenuates Kidney Injury in SI-AKI Mice

**Figure 2:**
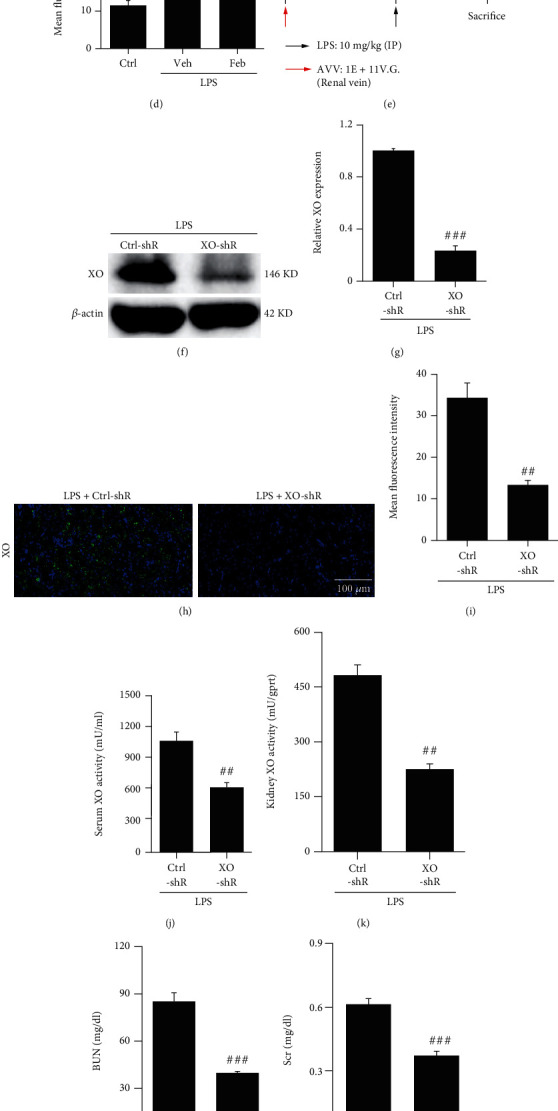
Febuxostat relieves serum and renal tissue XO activity, and XO knockdown attenuates kidney injury in SI-AKI mice. (a, b) The XO activity in serum and renal tissue homogenates was measured 24 h after LPS injection. XO could oxidize xanthine and produce O_2_^●^¯, which could react with WST-8, and the color of the reaction products could be detected with a micro reader at 450 nm. (c, d) Changes in XO expression in the kidneys of SI-AKI mice were detected by immunofluorescence assay (400x magnification). (e) Timeline of AAV injection displayed. (f–i) Knockdown of XO expression was confirmed using western blot and immunofluorescence assays. (j, k) Serum and renal XO activity were evaluated. (l, m) BUN and Scr were analyzed after downregulation of XO and challenge with LPS (10 mg/kg). (n, o) Representative images of kidney tissue are presented, and the injury score was graded in a double-blinded manner. ^∗^*P* < 0.05, ^∗∗^*P* < 0.01, and ^∗∗∗^*P* < 0.001 vs. Ctrl; ^#^*P* < 0.05, ^##^*P* < 0.01, and ^###^*P* < 0.001 vs. LPS+Veh or LPS+Ctrl-shR (*n* = 10). Ctrl: control; Veh: vehicle; LPS: lipopolysaccharide; Feb: febuxostat; XO: xanthine oxidase; BUN: blood urea nitrogen; Scr: serum creatinine.

Although febuxostat has shown an inhibitory effect on XO activity in other kidney injury models, this effect has not been reported in SI-AKI animal models. Consistent with the changes in renal function and histology, SI-AKI mice displayed higher XO activity in the serum and kidneys than control mice, and the increase in XO activity induced by LPS was reversed by febuxostat pretreatment (Figures 2(a) and 2(b)). Immunofluorescence assays further confirmed that the increased expression of XO in the kidney induced by LPS was inhibited by febuxostat pretreatment (Figures 2(c) and 2(d)). To further confirm the role of XO in SI-AKI, we constructed an AAV vector with XO shRNA (pAAV-shXO), and administration of pAAV-shXO significantly inhibited XO expression in the kidney (Figures 2(e)–2(i)). Serum and kidney XO activity were also decreased by XO knockdown (Figures 2(j) and 2(k)). Moreover, renal function, as indicated by BUN and Scr levels, was protected from the marked increase induced by XO knockdown in SI-AKI mice (Figures 2(l) and 2(m)). HE staining of kidney tissues from each group showed that XO knockdown dampened kidney tubular injury in SI-AKI mice (Figures 2(n) and 2(o)).

### 3.3. The Inhibition of XO Improves Hypoxia and ROS Production in the Kidneys of SI-AKI Mice

**Figure 3:**
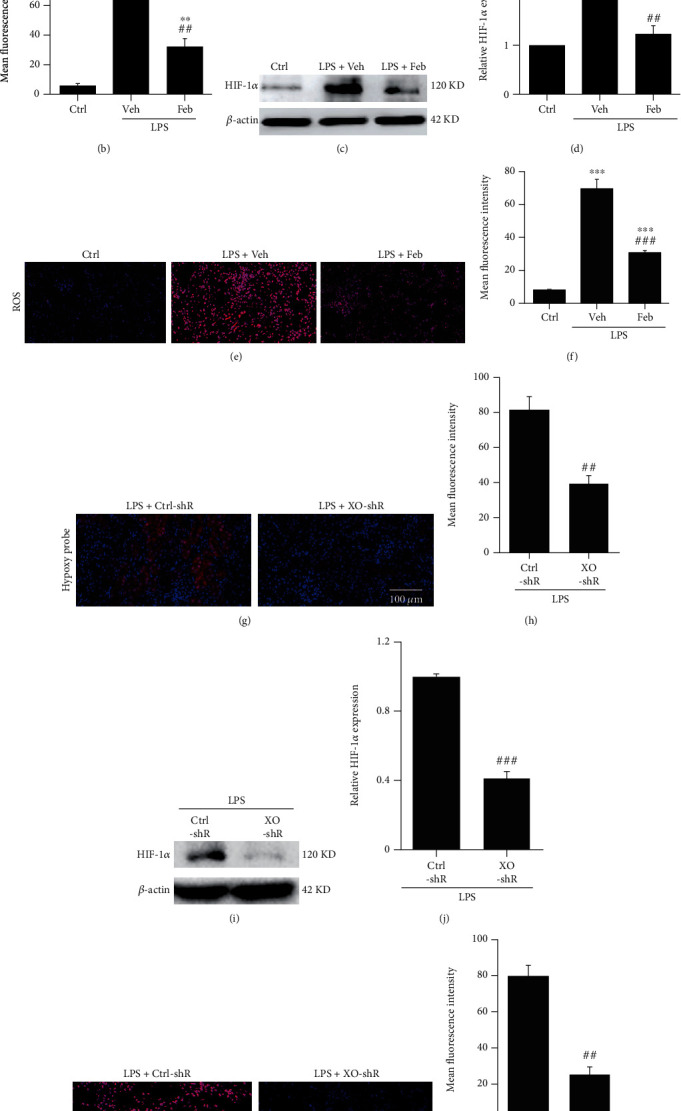
The inhibition of XO improves hypoxia and ROS production in the kidneys of SI-AKI mice. (a, b) Intracellular hypoxia in the kidneys of SI-AKI mice was detected by an immunofluorescence assay using a hypoxia probe (400x magnification). (c, d) Detection of HIF-1*α* protein expression in mouse renal homogenates by western blot. (e, f) The ROS level of kidney tissues was captured by CLSM at 24 h after LPS injection (dihydroethidium fluorescent staining, 400x magnification). (g, h) Intracellular hypoxia in the kidneys of SI-AKI mice after the knockdown of XO was captured by CLSM (400x magnification). (i, j) HIF-1*α* protein expression in SI-AKI mouse renal homogenates after downregulation of XO by western blot. (k, l) ROS levels in SI-AKI mouse renal tissue after the knockdown of XO were assessed using CLSM. Scale bar = 100 *μ*m. ^∗^*P* < 0.05, ^∗∗^*P* < 0.01, and ^∗∗∗^*P* < 0.001 vs. control; ^#^*P* < 0.05, ^##^*P* < 0.01, and ^###^*P* < 0.001 vs. LPS+Veh or LPS+Ctrl-shR (*n* = 10). Ctrl: control; Veh: vehicle; LPS: lipopolysaccharide; Feb: febuxostat; HIF: hypoxia-inducible factor; ROS: reactive oxygen species.

XO is an enzyme that utilizes molecular oxygen to produce ROS. Still it is unclear whether the inhibition of XO can improve renal hypoxia in SI-AKI mice by reducing the utilization of molecular oxygen. The hypoxia distribution in renal tubular cells can be detected by fluorescence labeling of the hypoxia probe pimonidazole, which can conjugate with intracellular thios under hypoxia and then be detected with a pimonidazole secondary antibody. The fluorescence intensity of the hypoxia signal in the kidney tissue of the SI-AKI group was the strongest, while that of the febuxostat pretreatment group was significantly decreased (Figures 3(a) and 3(b)). We also detected the expression of HIF-1*α* in the kidneys of SI-AKI mice. The results showed that the expression of HIF-1*α* in the kidneys of SI-AKI mice was significantly higher than that in control mice. At the same time, febuxostat pretreatment decreased the expression of HIF-1*α* in the kidneys of SI-AKI mice (Figures 3(c) and 3(d)). In addition, DHE fluorescence staining showed that febuxostat pretreatment significantly decreased the level of ROS in the kidneys of LPS-induced SI-AKI mice (Figures 3(e) and 3(f)). We further investigated the impact of XO on hypoxia and ROS in SI-AKI mice by knocking down XO in the kidney using pAAV-shXO. Downregulation of XO improved severe hypoxic conditions (Figures 3(g) and 3(h)) in SI-AKI mice and inhibited HIF-1*α* expression in the kidney (Figures 3(i) and 3(j)). The ROS level was also decreased by XO knockdown (Figures 3(k) and 3(l)).

### 3.4. Hypoxia Increased LPS-Induced XO Activity in HK-2 Cells, but the Inhibition of XO Conversely Improved Hypoxia

**Figure 4:**
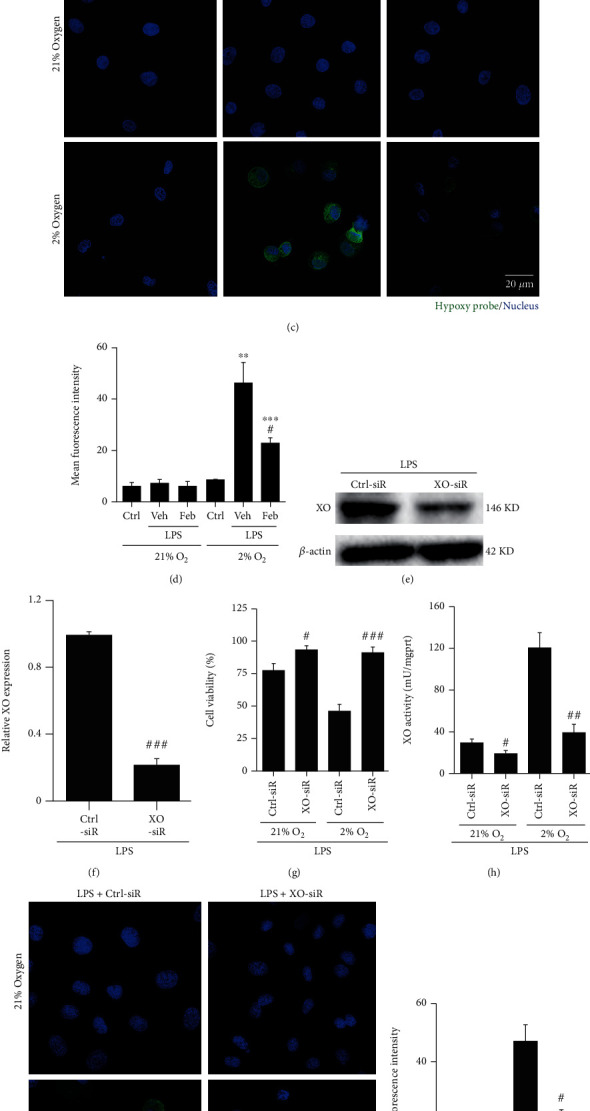
Hypoxia increased LPS-induced XO activity in HK-2 cells, but the inhibition of XO also improved hypoxia. (a) CCK8 showing the viability of HK-2 cells treated with LPS (10 *μ*g/ml)±febuxostat (100 *μ*M) under normoxic (21% O_2_) and hypoxic (2% O_2_) conditions for 6 h. (b) The XO activity of HK-2 cells treated with LPS±febuxostat under normoxic and hypoxic conditions for 6 h. (c, d) The intracellular hypoxia of HK-2 cells treated with LPS±febuxostat under normoxic and hypoxic conditions for 6 h was detected by an immunofluorescence assay using a hypoxia probe (800x magnification). (e, f) Knockdown of XO in HK-2 cells was confirmed using western blotting. (g) After the knockdown of XO with siRNA transfection, HK-2 cells were stimulated with LPS with 21% or 2% oxygen. Six hours later, the cells were harvested, and cell viability was assessed with the CCK8 method. (h) XO activity was assayed with the same method as in (b). (i, j) The intracellular hypoxia of HK-2 cells treated with LPS under normoxic and hypoxic conditions for 6 h was detected by an immunofluorescence assay using a hypoxia probe (800x magnification). Scale bar = 20 *μ*m. ^∗^*P* < 0.05, ^∗∗^*P* < 0.01, and ^∗∗∗^*P* < 0.001 vs. control; ^#^*P* < 0.05, ^##^*P* < 0.01, and ^###^*P* < 0.001 vs. LPS+Veh or LPS+Ctr-siR (*n* = 10). Ctrl: control; Veh: vehicle; LPS: lipopolysaccharide; Feb: febuxostat; XO: xanthine oxidase.

Since we observed the protective effect of febuxostat against renal hypoxia in SI-AKI mice in vivo, we further evaluated the relationship between XO activity and cellular hypoxia and the role of febuxostat. For this purpose, HK-2 cells were treated with LPS (10 *μ*g/ml)±febuxostat (100 *μ*M) and cultured under normoxic (21% O_2_, 5% CO_2_, and 74% N_2_) or hypoxic (2% O_2_, 5% CO_2_, and 93% N_2_) conditions for 6 h in vitro. The results from the cell counting kit-8 (CCK-8) assay showed that hypoxia for 6 h did not change the viability of HK-2 cells, which was decreased by LPS, especially under the condition of hypoxic culture (Figure 4(a)). The above results indicated that febuxostat reversed the increase in LPS-induced cytotoxicity under normoxia and hypoxia. Consistent with the results of CCK-8, LPS induced a slight increase in XO activity in HK-2 cells under normoxia and a significant increase in hypoxia, suggesting that hypoxia has a positive effect on the increase in XO activity induced by LPS (Figure 4(b)). Febuxostat inhibited the increase in XO activity induced by LPS under both normoxic and hypoxic conditions (Figure 4(b)). Importantly, the green fluorescence of the hypoxia probe showed that no obvious hypoxia occurred in HK-2 cells cultured in 21% oxygen with or without LPS±febuxostat treatment (Figures 4(c) and 4(d)). After culturing with 2% O_2_ for 6 h, HK-2 cells showed weak green fluorescence but LPS significantly enhanced the green fluorescence brightness, while febuxostat significantly reduced LPS-induced hypoxia (Figures 4(c) and 4(d)). To further confirm the function of XO in hypoxia- and LPS-induced cell injury, we knocked down XO in vitro using siRNA (Figures 4(e) and 4(f)). Loss of XO also alleviated hypoxia- and LPS-induced cell injury (Figure 4(g)) and XO activity (Figure 4(h)) and further relieved hypoxia in HK-2 cells (Figures 4(i) and 4(j)).

### 3.5. The Inhibition of XO Reduced Inflammation and Apoptosis in SI-AKI Mice

**Figure 5:**
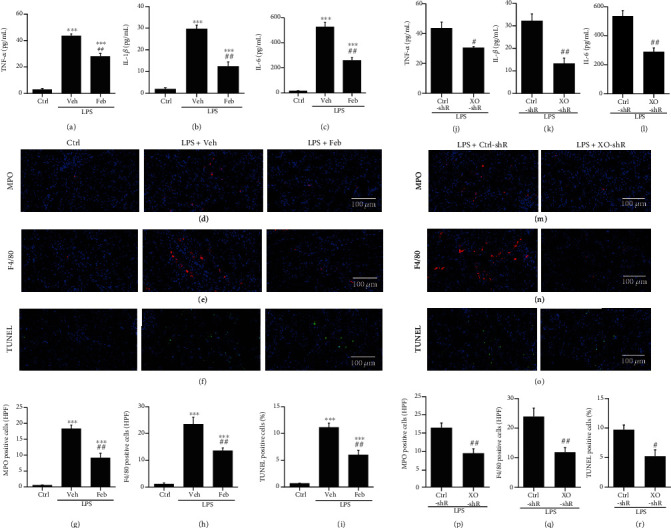
The inhibition of XO reduced inflammation and apoptosis in SI-AKI mice. (a–c) The levels of TNF-*α*, IL-1*β*, and IL-6 in renal homogenates of SI-AKI mice were detected by ELISA. (d, g) Immunofluorescence detection of neutrophils in the kidneys of SI-AKI mice by anti-MPO antibody (400x magnification). (e, h) Immunofluorescence detection of macrophages in the kidneys of SI-AKI mice by anti-F4/80 antibody (400x magnification). (f, i) Representative TUNEL-stained sections of the kidney in SI-AKI mice (400x magnification). Semiquantitative analysis of TUNEL-positive cells in each group is also displayed. (j–l) After the downregulation of XO, mice were treated with LPS (10 mg/kg), and the levels of TNF-*α*, IL-1*β*, and IL-6 in renal homogenates of SI-AKI mice were detected by ELISA. (m and p, n and q) After knockdown of XO with pAAV-shRNA, immunofluorescence detection of neutrophils and macrophages in the kidneys of SI-AKI mice by anti-MPO and anti-F4/80 antibodies (400x magnification). (o, r) Kidney cell apoptosis was analyzed with TUNEL staining in SI-AKI mice after XO knockdown. Scale bar = 100 *μ*m. ^∗^*P* < 0.05, ^∗∗^*P* < 0.01, and ^∗∗∗^*P* < 0.001 vs. control; ^#^*P* < 0.05, ^##^*P* < 0.01, and ^###^*P* < 0.001 vs. LPS+Veh or LPS+Ctr-shR (*n* = 10). Ctrl: control; Veh; vehicle; LPS: lipopolysaccharide; Feb: febuxostat; TNF: tumor necrosis factor; IL: interleukin; MPO: myeloperoxidase; TUNEL: terminal deoxynucleotidyl transferase dUTP nick-end labeling.

Hypoxia and oxidative stress induce mitochondrial damage, which further leads to the release of inflammatory cytokines and subsequent cell death [10]. Therefore, we further focused on the effects of XO inhibition on inflammation and apoptosis in SI-AKI mice. The ELISA results showed that febuxostat pretreatment significantly reduced the levels of serum inflammatory factors such as TNF-*α* (Figure 5(a)), IL-1*β* (Figure 5(b)), and IL-6 (Figure 5(c)) in SI-AKI mice triggered by LPS. Immunofluorescence detection of neutrophils and macrophages in renal tissue labeled with MPO (Figures 5(d) and 5(g)) and F4/80 (Figures 5(e) and 5(h)), respectively, showed that the infiltration of neutrophils and macrophages at the outer stripe of the outer medulla of the kidneys in SI-AKI mice increased significantly. Febuxostat pretreatment remarkably reduced neutrophil and macrophage infiltration. TUNEL assay in the kidneys indicated that febuxostat pretreatment significantly decreased the number of apoptotic cells induced by LPS (Figures 5(f) and 5(i)). We further investigated the impact of XO on inflammation and cell apoptosis in SI-AKI mice by knocking down XO in the kidney using pAAV-shXO. Downregulation of XO reduced the levels of serum TNF-*α* (Figure 5(j)), IL-1*β* (Figure 5(k)), and IL-6 (Figure 5(l)) in SI-AKI mice. Furthermore, neutrophil (Figures 5(m) and 5(p)) and macrophage (Figures 5(n) and 5(q)) infiltration was also reduced by XO knockdown. Cell apoptosis in the kidney was prevented by XO downregulation, as suggested by TUNEL staining (Figures 5(o) and 5(r)).

## 4. Discussion

Lipopolysaccharide (LPS), which is a component of the outer membrane of gram-negative bacteria, has been the most widely studied pathogen-associated molecular pattern (PAMP) in sepsis [28, 29]. LPS activates the Toll-like receptor to activate XO, which utilizes oxygen as a substrate to decompose hypoxanthine and xanthine into uric acid, producing superoxide and hydrogen peroxide during the reaction, and is mainly expressed during cellular stress or immune activation [30–32]. Hypoxemia caused by hemodynamic changes in sepsis can also activate XO, which is widespread in ischemia–reperfusion models [8, 17, 20, 21, 33]. Several experimental and clinical studies have proven that XO activity has proinflammatory and prooxidative effects and can mediate vascular and endothelial dysfunction. The inhibition of XO by allopurinol or Feb has a protective effect [14, 15, 17–21, 33, 34]. In this study, we found that febuxostat, an XO-specific inhibitor, and knockdown of XO expression with pAAV-shXO showed antioxidant stress and anti-inflammatory effects and weakened the local hypoxia of renal tubular epithelial cells by inhibiting XO activity, thus alleviating SI-AKI. The results of inhibition of XO in HK-2 cells with febuxostat and XO-siRNA in vitro showed that the downregulation of XO remarkably reduced the hypoxia condition in HK-2 cells induced by LPS and 2% oxygen. Taken together, our results indicated important roles of XO in oxidative stress- and hypoxia-induced injury in SI-AKI.

Currently, XO inhibitors approved by the US Food and Drug Administration (FDA) include three drugs, allopurinol, febuxostat, and topiroxostat, which all show antioxidant, anti-inflammatory, and renoprotective effects in addition to reducing uric acid [33]. Moreover, allopurinol and febuxostat have been confirmed to play an important role in myocardial mechanoenergetic uncoupling. Febuxostat is superior to allopurinol in reducing systolic blood pressure, pulse-wave velocity, and left ventricular mass index in hyperuricemic patients undergoing cardiac surgery [35]. Moreover, febuxostat was more effective and faster than allopurinol in achieving the serum uric acid target in patients with gout [36]. Similarly, febuxostat has an advantage over topiroxostat in cardiorenal protection in hyperuricemic patients with cardiovascular disease [37, 38]. Recently, there have been only two reports on the treatment of SI-AKI with febuxostat showing that febuxostat improves the prognosis of SI-AKI animals through antioxidant stress and anti-inflammation [20, 21]. In this study, we also confirmed that pretreatment with febuxostat or kidney knockdown of XO by shRNA in vivo significantly improved the prognosis of SI-AKI mice by reducing the levels of BUN, Scr, TNF-*α*, IL-6, and IL-1*β* in peripheral blood and by improving histological damage, reducing kidney tubular cell apoptosis and ROS production, and inhibiting infiltration of neutrophils and macrophages in the kidneys. This suggests that the inhibition of XO has a nephroprotective effect on SI-AKI through anti-inflammation, antioxidant stress, and antiapoptosis.

Sepsis is often accompanied by organ dysfunction, poor tissue perfusion, or hypotension, which is bound to ischemia/hypoxia of damaged organs [2–6]. Renal dysfunction in sepsis is usually secondary to septic shock and sometimes to hypovolemia [39, 40]. AKI, including endotoxemia, can reduce tissue oxygen delivery and increase renal tissue oxygen consumption, resulting in renal medullary hypoxia, which can be the main driver of a cascade of events leading to renal tubular dysfunction, vascular injury, and cell injury [41]. This study found that LPS induced significant hypoxia in renal tubular epithelial cells through hypoxia probe fluorescence detection. However, pretreatment with febuxostat or kidney knockdown of XO by shRNA in vivo significantly improved renal hypoxia in SI-AKI mice, suggesting that the inhibition of XO elicits a favorable effect on alleviating renal medullary hypoxia in SI-AKI mice. We further evaluated the effect of pharmacologic (febuxostat) and genetic (XO siRNA) inhibition on XO activity and the viability of HK-2 cells under LPS and hypoxia in vitro. Our results showed that LPS caused a slight increase in XO activity under normoxia and a significant increase under hypoxia, accompanied by consistent changes in the degree of hypoxia, suggesting that the effect of LPS on XO activity is closely related to cell hypoxia. Importantly, for HK-2 cells treated with LPS, pharmacologic and genetic inhibition of XO improved cell hypoxia at 2% oxygen concentration and reversed the decrease in cell viability induced by LPS, suggesting the positive effect of inhibition of XO in improving cell hypoxia.

Hypoxia-inducible factor (HIF) is a cellular oxygen sensor that is a heterodimeric protein composed of an *α* subunit and a *β* subunit [42]. Under normoxia, the *α* subunit is unstable, degraded by the ubiquitin–proteasome system and does not function [42]. Under hypoxia, stabilization of the *α* subunit results in the formation of a dimer with the *β* subunit, which translocates to the nucleus, initiating mRNA synthesis for multiple genes [42, 43]. For example, it can upregulate erythropoietin and endothelial nitric oxide synthase (eNOS) and ameliorate tissue hypoxia [22]. However, HIF can be a double-edged sword because early onset of renal medullary hypoxia in sepsis prolongs the phases of tissue hypoxia, leading to the destabilization of HIF that aggravates oxidative and nitrosative injuries, culminating in AKI [44]. In addition, excessive production of HIF in response to prolonged hypoxia in severe sepsis can lead to excessive production of vasoconstrictive and ROS-induced proteins, such as inducible nitric oxide synthase (iNOS), thereby promoting fibrogenesis [22]. Although our study did not further explore the advantages and disadvantages of high expression of HIF-1*α* in the kidneys of SI-AKI mice, the decrease in HIF-1*α* expression in the kidneys of mice with pharmacological and genetic inhibition of XO did not rule out the improvement of cellular hypoxia increased the degradation of HIF-1*α*, which further enriched the effect of XO inhibition in improving renal medulla hypoxia.

## 5. Conclusions

In summary, we proposed for the first time that the nephroprotective effect of inhibiting XO in SI-AKI models occurred at least partly through inhibiting XO activity to reduce renal hypoxia, thereby decreasing oxidative stress, inflammation, and apoptosis and ultimately attenuating the pathological process of SI-AKI. Although the specific regulatory pathway of XO inhibition to improve cellular hypoxia has still not been comprehensively illuminated, the current work might provide novel insight into the choice of drugs for the prevention and treatment of SI-AKI.

## Data Availability

All data related to this paper may also be requested from the corresponding authors (email: xjsnlhb@fmmu.edu.cn).

## Abbreviations

AKI: Acute kidney injury
SI-AKI: Sepsis-induced AKI
LPS: Lipopolysaccharide
Feb: Febuxostat
ROS: Reactive oxygen species
XO: Xanthine oxidase
CKD: Chronic kidney disease
BUN: Blood urea nitrogen
Scr: Serum creatinine
HIF: Hypoxia-inducible factor
DHE: Dihydroethidium
CLSM: Confocal laser-scanning microscope
MPO: Myeloperoxidase
TNF: Tumor necrosis factor
IL: Interleukin
TUNEL: Terminal deoxynucleotidyl transferase dUTP nick-end labeling.
